# The Effects of Immersive Virtual Reality–Assisted Experiential Learning on Enhancing Empathy in Undergraduate Health Care Students Toward Older Adults With Cognitive Impairment: Multiple-Methods Study

**DOI:** 10.2196/48566

**Published:** 2024-02-15

**Authors:** Justina Yat Wa Liu, Pui Ying Mak, Kitty Chan, Daphne Sze Ki Cheung, Kin Cheung, Kenneth N K Fong, Patrick Pui Kin Kor, Timothy Kam Hung Lai, Tulio Maximo

**Affiliations:** 1 School of Nursing The Hong Kong Polytechnic University Hong Kong China (Hong Kong); 2 Research Institute for Smart Ageing The Hong Kong Polytechnic University Hong Kong China (Hong Kong); 3 Department of Rehabilitation Sciences The Hong Kong Polytechnic University Hong Kong China (Hong Kong); 4 School of Design The Hong Kong Polytechnic University Hong Kong China (Hong Kong)

**Keywords:** immersive virtual reality, undergraduate health care education, empathy, cognitive impairment

## Abstract

**Background:**

Immersive virtual reality (IVR)–assisted experiential learning has the potential to foster empathy among undergraduate health care students toward older adults with cognitive impairment by facilitating a sense of embodiment. However, the extent of its effectiveness, including enhancing students’ learning experiences and achieving intended learning outcomes, remains underexplored.

**Objective:**

This study aims to evaluate the impacts of IVR-assisted experiential learning on the empathy of undergraduate health care students toward older people with cognitive impairment as the primary outcome (objective 1) and on their learning experience (objective 2) and their attainment of learning outcomes as the secondary outcomes (objective 3).

**Methods:**

A multiple-methods design was used, which included surveys, focus groups, and a review of the students’ group assignments. Survey data were summarized using descriptive statistics, whereas paired 2-tailed *t* tests were used to evaluate differences in empathy scores before and after the 2-hour IVR tutorial (objective 1). Focus groups were conducted to evaluate the impacts of IVR-assisted experiential learning on the empathy of undergraduate health care students toward older people with cognitive impairment (objective 1). Descriptive statistics obtained from surveys and thematic analyses of focus groups were used to explore the students’ learning experiences (objective 2). Thematic analysis of group assignments was conducted to identify learning outcomes (objective 3).

**Results:**

A total of 367 undergraduate nursing and occupational therapy students were recruited via convenience sampling. There was a significant increase in the students’ empathy scores, measured using the Kiersma-Chen Empathy Scale, from 78.06 (SD 7.72) before to 81.17 (SD 8.93) after (*P*<.001). Students expressed high satisfaction with the IVR learning innovation, with a high satisfaction mean score of 20.68 (SD 2.55) and a high self-confidence mean score of 32.04 (SD 3.52) on the Student Satisfaction and Self-Confidence scale. Students exhibited a good sense of presence in the IVR learning environment, as reflected in the scores for adaptation (41.30, SD 6.03), interface quality (11.36, SD 3.70), involvement (62.00, SD 9.47), and sensory fidelity (31.47, SD 5.23) on the Presence Questionnaire version 2.0. In total, 3 major themes were identified from the focus groups, which involved 23 nursing students: *enhanced sympathy toward older adults with cognitive impairment*, *improved engagement in IVR learning*, and *confidence in understanding the key concepts through the learning process*. These themes supplement and align with the survey results. The analysis of the written assignments revealed that students attained the learning outcomes of understanding the challenges faced by older adults with cognitive impairment, the importance of providing person-centered care, and the need for an age-friendly society.

**Conclusions:**

IVR-assisted experiential learning enhances students’ knowledge and empathy in caring for older adults with cognitive impairment. These findings suggest that IVR can be a valuable tool in professional health care education.

## Introduction

### Background

Empathy is a cognitive ability that involves understanding other people’s experiences, concerns, and perspectives, along with a capacity to communicate this understanding and the motivation to help others [1,2]. Showing empathy to patients, such as through active listening and self-awareness, is associated with improved patient outcomes and satisfaction [3,4]. When health care professionals understand the needs of patients, patients may feel more secure in relating their concerns to health care professionals and raising issues that worry them [5].

Although the Association of American Medical Colleges identifies empathy as an essential learning objective in health care education [6], undergraduate health care students have been found to have negative attitudes toward older people, affecting their willingness to work in this specialty [7-10]. This is especially true for older adults with cognitive impairment, about whom undergraduate health care students may hold stereotypes and whom they might socially stigmatize, leading to concerns about a possible lack of attentiveness in the provision of care to this group [11].

Empathy has been found to be positively correlated with the attitude of undergraduate health care students toward older adults and their willingness to care for them [12,13]. The most common methods for cultivating empathy in students include experiential training, didactic training, skills training, and a mixed methods approach [14]. Experiential learning is cognitively stimulating and has an impact on the entire person. It allows students to acquire knowledge, skills, and attitudes cognitively, affectively, and behaviorally [15]. Undergraduate health care students can benefit from experiential learning by considering the perspectives of the patients and experiencing them firsthand [16]. Experiential learning allows undergraduate health care students to gain more insights into how to solve the problems that older adults with cognitive impairment may encounter [17]. It is usually challenging for undergraduate health care students to understand the needs of older adults with cognitive impairment as these older adults may not be able to clearly communicate their needs [18]. However, through experiential learning, students can gain hands-on experiences that can give them a deeper knowledge and understanding of the challenges that older adults with cognitive impairment may be encountering [19].

Despite being suitable for enhancing empathy in undergraduate health care students, the various forms of conventional experiential learning, including service learning, role-play, and simulation-based workshops, have limitations in terms of replicating realistic scenarios and patients in an authentic environment. In addition, in situations in which students may become distracted, instruction from supervisors is always required [20]. For example, in role-play, not all students can immerse themselves in the role of the patient [21], affecting their learning experience. However, a new type of experiential learning delivered via immersive virtual reality (IVR) provides students with an environment that encompasses them perceptually and gives them the feeling of being within it [22]. Owing to IVR’s capacity to stimulate different senses concurrently, it is highly efficient in immersing users and generating a strong sense of presence. It is becoming more common to use IVR in health care education. However, there is a scarcity of research on such IVR experiences in an educational context [23].

IVR provides students with a realistic but safe virtual clinical environment, allowing them to gain insights into patients’ perspectives through their eyes, voices, and emotions [24]. Buchman and Henderson [25] reported that undergraduate health care students had enhanced empathy and felt a sense of realism and authenticity in the IVR experience, with empathy being the clear theme arising from the focus group analysis. Undergraduate health care students have undoubtedly also reported positive experiences with receiving different types of experiential learning other than IVR [26]. However, the sense of presence and realism generated from IVR is not possible in conventional experiential learning. IVR-assisted experiential learning is also a highly customized learning method targeted at achieving specific learning outcomes [27]. By using IVR, teachers can put undergraduate health care students in situations that are tailored to their learning needs and outcomes, whereas this level of customization may be challenging to attain in conventional experiential learning, which invariably uses a one-size-fits-all approach. Nursing students have also been found to have a higher level of engagement when taking part in IVR learning compared with their engagement with conventional learning methods, and teachers have found IVR to be helpful in compensating for the limited clinical placements available for students in hospitals [28].

Previous studies have recognized the effectiveness of IVR-assisted experiential learning in improving empathy among undergraduate health care students [29]. The Cognitive Affective Model of Immersive Learning by Makransky and Petersen [30] suggests that the mental state of perceiving a virtual self as one’s actual self with a heightened sense of embodiment refers to the sensation of possessing a virtual body. Hence, using a first-person viewpoint with a virtual environment through IVR as a “perspective taking machine” could lead to a feeling of immersion and improve a participant’s level of embodiment, leading to an increase in empathy [31-33]. Scholars have also recommended that medical students participate in IVR experiential learning to improve their empathy before starting their clinical placement [34].

Despite previous studies, there has been little discussion on whether IVR-assisted experiential learning can enhance students’ attainment of learning outcomes such as understanding the special needs of older adults with cognitive impairment. Although there has been one study examining the improvement in the cognitive skills, such as communication competency, of multidisciplinary undergraduate and graduate health care students after an IVR simulation, its findings were based on the self-perceived evaluation of students [35]. This approach appears to lack a comparatively objective way of measuring learning outcomes, and the results of the study may be inconclusive as they may not reflect actual learning outcomes. To address this knowledge gap, it may be necessary to place more emphasis on comparatively objective assessments, such as teacher evaluations conducted according to preset assessment rubrics related to the learning outcomes.

### Objectives

Therefore, this study aimed not only to evaluate the effects of IVR-assisted experiential learning on enhancing the empathy of undergraduate health care students toward older people with cognitive impairment (objective 1) but also to explore the students’ learning experiences, including “students’ satisfaction and self-confidence in learning” and “IVR fidelity” (objective 2), and their learning outcomes (objective 3) after attending the IVR-assisted experiential tutorial.

## Methods

A multiple-methods design was used, which included a survey, focus groups, and student assignment reviews [36], to assess the effectiveness of the IVR-assisted experiential tutorial on students’ empathy and learning experiences and outcomes. This design produces more comprehensive findings than those obtained in single-method studies [37].

### Participants

Convenience sampling was used to recruit participants for this study. Specifically, those invited to participate were undergraduate year-3 nursing students (n=267) who were taking the subject of gerontological nursing and year-3 occupational therapy (OT) students (n=100) who were taking the subject of human occupations. The nursing students were divided into 33 groups of 7 to 8 students each. They were invited to send a representative to participate in the focus groups. Ultimately, 23 group representatives participated in the focus groups. As a required learning activity, all students were obligated to attend the tutorial. However, they were given the option to join the study and complete surveys to share their learning experiences with the research team, of which 3 members (JYWL, PPKK, and KNKF) were subject lecturers. Only those who consented to join the study were included in the analysis and reporting of the results, and their anonymity was maintained in this paper.

### Design of the IVR-Assisted Experiential Tutorial

#### Overview

To ensure that students had a solid grasp of the foundational knowledge in the subjects of gerontological nursing (for nursing students) and human occupations (for OT students), a 2-hour IVR-assisted experiential tutorial was arranged in week 7, halfway through the 13-week courses. Only the nursing students were mandated to complete and submit a group assignment within 2 weeks following the IVR tutorial.

**Figure 1:**
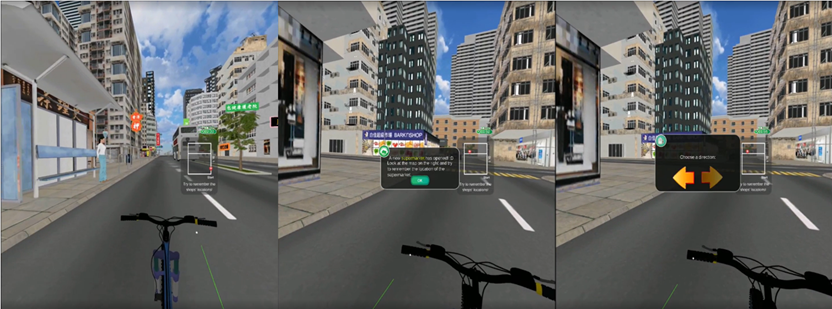
Scenarios simulating getting lost when looking for a supermarket as experienced by individuals with cognitive impairment.

**Figure 2:**
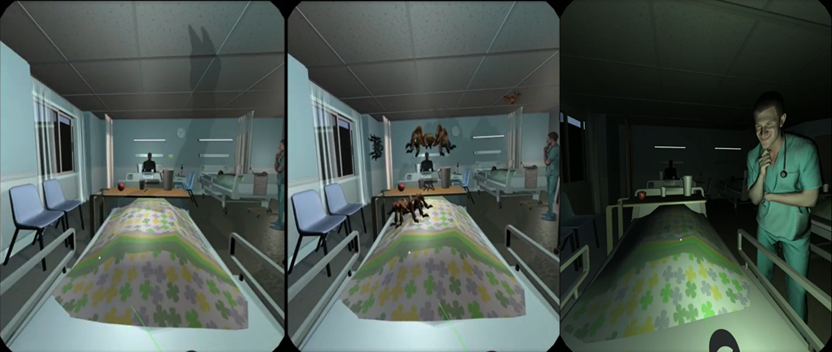
Scenarios simulating the hallucinations experienced by older adults with delirium.

The research team developed 2 IVR games that simulated experiences commonly encountered by older adults with cognitive impairment. The first IVR game simulated a scenario in which an individual with cognitive impairment gets lost in a community setting (Figure 1). The second IVR game simulated the distorted auditory and visual perceptions commonly experienced by older adults with delirium (Figure 2). These are common challenges faced on a daily basis by older adults with cognitive impairments. These 2 IVR games were used in the 2-hour IVR-assisted experiential tutorial. Each tutorial comprised students aged between 25 and 30 years who were divided into 7 to 8 subgroups. Each subgroup underwent concurrent IVR–assisted experiential learning.

The intended learning outcomes of the IVR-assisted experiential learning tutorial were as follows: (1) students would gain insights into the lives of older adults with cognitive impairment and their problem-solving efforts when facing daily challenges and, thus, develop empathy toward this group of older adults, (2) students would apply the skills and knowledge that they learned about common situations to propose more inclusive solutions targeted at older adults with cognitive impairment, and (3) students would be able to develop age-friendly care plans to meet the whole-person needs of older adults with cognitive impairment.

On the basis of the experiential learning model suggested by Kolb [38], 4 stages were included in the tutorial to enhance the students’ learning experiences and outcomes.

#### Stage 1: Concrete Experience Through Experiential Learning

The students’ concrete experience was obtained by exposing them to 10 to 15 minutes of IVR environments through head-mounted devices. This involved creating a realistic and immersive virtual environment that simulated a real-world experience, allowing students to engage with the internet-based environment in a meaningful way. For example, students were required to complete some daily tasks (eg, finding a supermarket) in the virtual reality (VR) environment while overwhelmed by stimuli to mimic the experiences of older people with cognitive impairment or during delirium, such as encountering confusing noises and images played through a VR head-mounted device.

#### Stages 2 and 3: Reflective Observations and Abstract Conceptualizations Through Reflective and Integrative Learning During Debriefings

Debriefing is considered an important element in experiential-based learning that reinforces and helps consolidate learning [39]. Reflective observation involves reflecting on the experience and considering what happened during the IVR simulation. The subject lecturers guided the students to reflect on and discuss the thoughts, feelings, and emotions that they experienced during the IVR-assisted experiential learning. This reflective process can help students gain insights into their own behavior and thought patterns as well as identify areas for improvement [40].

Abstract conceptualization involves interpreting and integrating the IVR experience into existing knowledge and understanding [41]. Therefore, students were motivated to reflect on and make connections between their previous experiences with older people and the insights that they gained from the IVR games. Through this process, the students showed that they were acquiring a deeper understanding of the complexities and challenges that older people with cognitive impairment face in everyday life. At the same time, students experienced the frustration and vulnerability associated with these challenges while navigating the IVR environment. The students became aware of the need for empathy, good communication, compassion, a caring and respectful attitude, and patience when working with older people with different impairments. This reflective and integrative learning approach helped cultivate empathy among the students and gave them a deeper understanding of the needs of older people.

#### Stage 4: Active Experimentation by Applying the Learning in Practical Ways

Afterward, each subtutorial group in the nursing subject was required to submit a written group report to describe the strategies (a plan) for assisting older people with cognitive impairment to remain in society. The students were expected to relate the knowledge and experiences they had gained from IVR experiential learning to the proposed strategies. They shared their strategies with their teachers and fellow students on Blackboard (a web-based education platform; Anthology Inc). The lecturers evaluated the students’ performance on this assignment based on the predeveloped rubric. This exercise in active experimentation equipped the students with the skills that they would need to work with older people and develop their advocacy roles in practice.

### Outcome Measures

#### Empathy Toward Older Adults (Objective 1)

Students’ empathy toward older adults (objective 1) was measured using the Kiersma-Chen Empathy Scale (KCES). The 15-item KCES was developed from the theoretical perspective of empathy, which includes cognitive (ie, the ability to understand and view the world from the perspective of other people) and affective (ie, the ability to connect with the experiences or feelings of others) aspects [42]. Each item in the KCES is rated on a 7-point Likert-type scale (1=*strongly disagree*; 7=*strongly agree*). The scores on the KCES range from 15 to 105, with higher scores indicating greater empathy toward older adults. The KCES has demonstrated good test-retest reliability, with an intraclass correlation coefficient of 0.78. It correlates positively with the Jefferson Scale of Physician Empathy [43] (*r*=0.52) and negatively with the cynicism subscale of the Maslach Burnout Inventory (*r*=−0.24) [44], providing evidence of its construct validity [42]. Students were asked to complete this web-based questionnaire 1 week before the VR-assisted experiential tutorial and return the posttest questionnaire within 1 week after the tutorial.

#### Learning Experience (Objective 2)

The students’ experiences in learning (objective 2) with IVR-assisted experiential learning were evaluated through a posttutorial web-based survey and a focus group interview. The Student Satisfaction and Self-Confidence scale was administered after the completion of the IVR experiential tutorial. This questionnaire contains 13 items with 2 subscales (ie, satisfaction and self-confidence). Each item is rated on a 5-point Likert scale ranging from 1 (*strongly disagree with the statement*) to 5 (*strongly agree with the statement*). The scores on the satisfaction with learning scale range from 5 to 25, and the self-confidence scores range from 8 to 40, with a higher score indicating greater satisfaction and self-confidence, respectively. Both scales had high internal reliability, with a Cronbach α of .94 and .87 for the satisfaction and self-confidence scales, respectively [45].

The Presence Questionnaire version 2.0 (PQ2) was also used to evaluate the students’ sense of presence in the IVR environments (ie, IVR fidelity; objective 2) [46,47] after the IVR-assisted experiential tutorial class. The 29-item questionnaire includes 4 subscales: involvement (score range from 0 to 84), sensory fidelity (score range from 0 to 42), adaption or immersion (score range from 0 to 56), and interface quality (score range from 0 to 21), with higher scores indicating better or higher involvement, sensory fidelity, adaption or immersion, and interface quality. The students rated their experiences on a 7-point Likert scale from 1 (*not at all*) to 7 (*completely*). The PQ2 has been found to have high internal consistency, with a Cronbach α coefficient of .90, and correlate strongly with other measures of presence (*r*=0.78) [46].

A trained research assistant conducted 3 focus groups, with each group comprising 7 to 8 nursing students, to explore their learning experiences (objective 2) with IVR. They were asked questions such as the following: “What was your overall experience with IVR in your learning?” “How did IVR contribute to your understanding of the daily challenges of older people with cognitive impairment?” “Did you face any challenges or difficulties while using IVR for learning?” “How did IVR compare to other learning methods?” and “What suggestions do you have for improving the use of IVR in learning?” The interviews were audio recorded and then transcribed verbatim.

#### Learning Outcomes (Objective 3)

In this study, the impact on the students’ attainment of the learning outcomes (objective 3) referred to the students’ ability to show their understanding of the needs of older people with cognitive impairment (intended learning outcome 1) and their ability to apply this knowledge to identify inclusive strategies to help older people stay in the community (intended learning outcome 2). Only nursing students were required to complete a group assignment to describe the plan and strategies to develop age-friendly care plans to meet older adults’ needs (intended learning outcome 3). The Design of the IVR-Assisted Experiential Tutorial section provides details on the intended learning outcomes of the tutorial, and the Stage 4: Active Experimentation section provides details on the arrangement of the assignment. The group assignment was evaluated based on the assessment rubric by the lecturers of gerontological nursing (JYWL and PPKK), who were also members of the project team.

### Data Analysis

The numerical data collected via the surveys were summarized as descriptive statistics using SPSS (version 27; IBM Corp) for the analysis. Simple frequencies, percentages, means, and SDs were calculated. For the pre- and posttest assessments, paired 2-tailed *t* tests and confidence levels were calculated to test the differences before and after the tutorial. The level of significance was set at *P*<.05, and all tests were 2-tailed.

The text data collected through focus groups to identify the students’ learning experiences were analyzed using descriptive thematic analysis. To identify the students’ achievement of the learning outcomes, their written assignments were also analyzed using a descriptive thematic analysis. In contrast to other similar approaches, in thematic analysis, there is no commitment to a specific theoretical framework; therefore, a thematic analysis can be used between various theoretical frameworks. Thus, it is a more accessible and flexible form of analysis. What researchers do with the themes once they are uncovered will differ based on the aim of the research and the process of analysis [48]. In total, 2 researchers (JYWL and PPKK) read the students’ written assignments and independently identified codes from them. Codes with similar content were grouped together to form subthemes. The subthemes were then categorized into themes. Another researcher (KC) reviewed the codes, subthemes, and themes, and any discrepancies were resolved through discussion to achieve a consensus.

### Ethical Considerations

This study was approved by the Human Subjects Ethics Application Review System of the Hong Kong Polytechnic University (HSEARS20200423001) and conducted between June 2021 and May 2022. It was carried out in accordance with the Declaration of Helsinki. This included but was not limited to guaranteeing the anonymity of participants and obtaining the informed consent of the participating students. The participation of the students was voluntary, and their academic results were not affected by their decision to participate in the study.

## Results

### Overview

Of the 367 students who were enrolled in the 2 subjects, 93.7% (344/367) consented to join the study, of whom 75.6% (260/344) were nursing students and 24.4% (84/344) were OT students. They completed and returned the pre- and posttest surveys with an overall response rate of 93.7% (344/367). Most participating students were female (256/344, 74.4%), 23.3% (80/344) were male, and 2.3% (8/344) did not report their gender. Their ages ranged from 18 to 24 years.

We invited all 33 subgroups from the nursing subject to send 1 representative to join the focus groups. Eventually, 23 group representatives (a response rate of 23/33, 70%) participated in the focus groups, of whom 16 (70%) were female students. The participants were assigned to 1 of the 3 focus groups, with each group comprising 7 to 8 students to facilitate in-depth group discussions.

### Empathy Toward Older Adults (Objective 1)

Participating students showed moderate empathy toward older people, as reflected by a KCES score of 78.06 (SD 7.72) out of 105 before the IVR-assisted experiential tutorial. After completing the tutorial, this score increased to 81.17 (SD 8.93). The results of the paired-sample 2-tailed *t* test showed a significant increase in the mean score from before to after the tutorial (*t*_304_=3.95; *P*<.001; Table 1). A further subgroup analysis was conducted, and a significant difference was found in the results between the nursing and OT students in KCES scores. There was a significant improvement in KCES scores among the nursing students but a decreasing trend among the OT students (Multimedia Appendix 1).

**Table 1:** Changes in the Kiersma-Chen Empathy Scale (KCES) score before and after immersive virtual reality experiential learning (n=344)

Question	Before, mean score (SD)	After, mean score (SD)	*t* statistic		
			Mean difference (SD)	*t* test (*df*)	*P* value
1. It is necessary for a health care practitioner to be able to comprehend someone else’s experiences.	5.80 (0.89)	5.83 (0.91)	0.03 (1.11)	0.46 (304)	.64
2. I am able to express my understanding of someone’s feelings.	5.38 (0.92)	5.61 (0.91)	0.23 (1.04)	3.91 (304)	<.001
3. I am able to comprehend someone else’s experiences.	5.33 (0.85)	5.65 (0.88)	0.32 (1.06)	5.32 (304)	<.001
4. I will not allow myself to be influenced by someone’s feelings when determining the best treatment^a^.	4.62 (1.27)	4.65 (1.43)	0.62 (2.28)	4.88 (304)	<.001
5. It is necessary for a health care practitioner to be able to express an understanding of someone’s feelings.	5.77 (0.85)	5.85 (0.74)	0.79 (0.93)	1.48 (304)	.14
6. It is necessary for a health care practitioner to be able to value someone else’s point of view.	5.80 (0.88)	5.93 (0.82)	0.13 (1.04)	2.10 (304)	.04
7. I believe that caring is essential to building a strong relationship with patients.	6.06 (0.79)	6.03 (0.82)	0.03 (0.89)	0.58 (304)	.56
8. I am able to view the world from another person’s perspective.	5.34 (0.94)	5.69 (0.85)	0.35 (1.15)	5.26 (304)	<.001
9. Considering someone’s feelings is not necessary to provide patient-centered care^a^.	3.15 (1.79)	3.78 (2.11)	0.64 (2.28)	4.88 (304)	<.001
10. I am able to value someone else’s point of view.	5.43 (0.87)	5.70 (0.84)	0.27 (1.05)	4.52 (304)	<.001
11. I have difficulty identifying with some else’s feelings^a^.	3.51 (1.43)	3.98 (1.66)	0.47 (1.88)	4.38 (304)	<.001
12. To build a strong relationship with patients, it is essential for a health care practitioner to be caring.	5.81 (0.88)	5.93 (0.82)	0.12 (1.02)	2.03 (304)	.04
13. It is necessary for a health care practitioner to identify with someone else’s feelings.	5.73 (0.87)	5.94 (0.79)	0.20 (0.94)	3.77 (304)	<.001
14. It is necessary for a health care practitioner to be able to view the world from another person’s perspective.	5.69 (0.87)	5.90 (0.84)	0.21 (0.92)	4.03 (304)	<.001
15. A health care practitioner should not be influenced by someone’s feelings when determining the best treatment^a^.	4.81 (1.36)	4.70 (1.60)	0.11 (1.59)	1.15 (304)	.25
Total KCES	78.06 (7.72)	81.17 (8.93)	3.11 (0.523)	3.95 (304)	<.001

^a^Items with negative wordings are scored in reverse.

### Learning Experience (Objective 2)

#### Students’ Satisfaction and Self-Confidence in Learning

Students were satisfied with the current learning innovation, as reflected by a high satisfaction mean score of 20.68 (SD 2.55) out of 25. For example, 92.7% (319/344) of the students agreed or strongly agreed that IVR-assisted experiential learning was suitable for the way they learned (item 5). The same percentage of students agreed or strongly agreed that the IVR learning experience provided an alternative learning experience to promote their learning interests (item 2). A total of 91.6% (315/344) of the students agreed or strongly agreed that the IVR simulation was motivating and helped them learn better (item 4).

They also showed a high level of self-confidence in their IVR experiential learning, with a mean score of 32.04 (SD 3.52) out of 40. Approximately 85.5% (294/344) of the students agreed or strongly agreed that they were confident that they would obtain the necessary skills and knowledge through learning with the IVR simulation (items 6-8). A total of 95.1% (327/344) of the participants agreed or strongly agreed that students should take responsibility for their learning (items 10-11; Table 2).

**Table 2:** The findings of the Student Satisfaction and Self-Confidence scale (n=344).

Item		Participants, n (%)				
		Strongly disagree (1)	Disagree (2)	Undecided (3)	Agree (4)	Strongly agree (5)
**Satisfaction with the current learning subscale**						
	1. The teaching methods used in the IVR^a^ simulation were helpful and effective.	2 (0.6)	4 (1.2)	20 (5.8)	242 (70.4)	76 (22.1)
	2. The IVR simulation provided me with a variety of learning materials and activities to promote my learning curriculum.	1 (0.3)	5 (1.5)	19 (5.5)	244 (70.9)	75 (21.8)
	3. I enjoyed how my instructor taught the IVR simulation.	1 (0.3)	4 (1.2)	25 (7.3)	233 (67.7)	81 (23.5)
	4. The teaching materials used in this IVR simulation were motivating and helped me to learn.	1 (0.3)	5 (1.5)	23 (6.7)	232 (67.4)	83 (24.1)
	5. The way my instructor taught the IVR simulation was suitable to the way I learn.	1 (0.3)	3 (0.9)	21 (6.1)	246 (71.5)	73 (21.2)
**Self-confidence in learning subscale**						
	6. I am confident that I am mastering the content of the IVR simulation activity that my instructor presented to me.	1 (0.3)	7 (2)	45 (13.1)	234 (68)	57 (16.6)
	7. I am confident that this simulation covered critical content necessary for the mastery of the curriculum.	1 (0.3)	6 (1.7)	47 (13.7)	239 (69.5)	51 (14.8)
	8. I am confident that I am developing the skills and obtaining the required knowledge from this simulation to perform necessary tasks in a clinical setting.	1 (0.3)	11 (3.2)	38 (11)	246 (71.5)	48 (14)
	9. My instructors used helpful resources to teach the simulation.	1 (0.3)	5 (1.5)	17 (4.9)	244 (70.9)	77 (22.4)
	10. It is my responsibility as the student to learn what I need to know from this IVR simulation activity.	1 (0.3)	1 (0.3)	15 (4.4)	260 (75.6)	67 (19.5)
	11. I know how to get help when I do not understand the concepts covered in the simulation.	1 (0.3)	6 (1.7)	28 (8.1)	254 (73.8)	55 (16)
	12. I know how to use simulation activities to learn critical aspects of these skills.	1 (0.3)	5 (1.5)	30 (8.7)	246 (71.5)	62 (18)
	13. It is the instructor’s responsibility to tell me what I need to learn of the simulation activity content during class time.	1 (0.3)	27 (7.8)	89 (25.9)	187 (54.4)	40 (11.6)

^a^IVR: immersive virtual reality.

#### IVR Fidelity

IVR fidelity was measured using the PQ2. The results showed that students developed a good sense of presence in the IVR learning environment, as seen in their scores on adaptation (mean 41.30, SD 6.03 out of 56), interface quality (mean 11.36, SD 3.70 out of 21), involvement (mean 62.0, SD 9.47 out of 84), and sensory fidelity (mean 31.47, SD 5.23 out of 42) (Table 3).

On the basis of the focus group discussions with the students about their experiences of experiential learning with IVR, 4 themes were identified: *enhanced sympathetic feeling toward older adults with cognitive impairment*, *improved engagement in IVR learning, confidence in understanding key concepts in the IVR experiential learning process,* and *limitations of IVR technology*.

**Table 3:** The findings of the Presence Questionnaire version 2.0 (n=344).

Item			Participants, n (%)												
			1		2		3		4		5		6		7
**Involvement**															
	1. How much were you able to control events?	3 (0.9)		4 (1.2)		3 (0.9)		45 (13.1)		73 (21.2)		156 (45.3)		60 (17.4)	
	2. How responsive was the environment to actions that you initiated (or performed)?	1 (0.3)		5 (1.5)		9 (2.6)		66 (19.2)		116 (33.7)		107 (31.1)		40 (11.6)	
	3. How natural did your interactions with the IVR^a^ environment seem?	8 (2.3)		6 (1.7)		23 (6.7)		64 (18.6)		121 (35.2)		92 (26.7)		30 (8.7)	
	4. How much did the visual aspects of the IVR environment involve you?	1 (0.3)		1 (0.3)		11 (3.2)		43 (12.5)		80 (23.3)		155 (45.1)		53 (15.4)	
	6. How natural was the mechanism that controlled movement through the environment?	6 (1.7)		7 (2)		26 (7.6)		48 (14)		134 (39)		99 (28.8)		24 (7)	
	7. How compelling was your sense of objects moving through space?	2 (0.6)		3 (0.9)		10 (2.9)		55 (16)		133 (38.7)		110 (32)		31 (9)	
	8. How much did your experiences in the virtual environment seem to be consistent with your real-world experiences?	14 (4.1)		13 (3.8)		24 (7)		69 (20.1)		109 (31.7)		93 (27)		22 (6.4)	
	10. How completely were you able to actively survey or search the IVR environment using vision?	1 (0.3)		3 (0.9)		6 (1.7)		41 (11.9)		132 (38.4)		121 (35.2)		40 (11.6)	
	14. How compelling was your sense of moving around inside the virtual environment?	1 (0.3)		4 (1.2)		11 (3.2)		60 (17.4)		140 (40.7)		96 (27.9)		32 (9.3)	
	17. How well could you move or manipulate objects in the virtual environment?	12 (3.5)		2 (0.6)		22 (6.4)		67 (19.5)		116 (33.7)		100 (29.1)		25 (7.3)	
	18. How involved were you in the virtual environment experience?	3 (0.9)		5 (1.5)		6 (1.7)		42 (12.2)		115 (33.4)		124 (36)		49 (14.2)	
	26. How easy was it to identify objects through physical interaction (eg, touching an object, walking over a surface, or bumping into a wall or object)?^b^	10 (2.9)		11 (3.2)		24 (7)		91 (26.5)		126 (36.6)		58 (16.9)		24 (7)	
**Sensory fidelity**															
	5. How much did the auditory aspects of the IVR environment involve you?	5 (1.5)		3 (0.9)		18 (5.2)		49 (14.2)		103 (29.9)		118 (34.3)		48 (14)	
	11. How well could you identify sounds?	3 (0.9)		4 (1.2)		8 (2.3)		43 (12.5)		107 (31.1)		127 (36.9)		52 (15.1)	
	12. How well could you localize sounds?	3 (0.9)		6 (1.7)		11 (3.2)		50 (14.5)		118 (34.3)		114 (33.1)		42 (12.2)	
	13. How well could you actively survey or search the virtual environment using touch?	10 (2.9)		9 (2.6)		21 (6.1)		57 (16.6)		120 (34.9)		98 (28.5)		29 (8.4)	
	15. How closely were you able to examine objects?	2 (0.6)		4 (1.2)		17 (4.9)		59 (17.2)		129 (37.5)		102 (29.7)		31 (9)	
	16. How well could you examine objects from multiple viewpoints?	1 (0.3)		2 (0.6)		12 (3.5)		64 (18.6)		118 (34.3)		116 (33.7)		31 (9)	
**Adaption or immersion**															
	9. Were you able to anticipate what would happen next in response to the actions that you performed?	4 (1.2)		9 (2.6)		39 (11.3)		63 (18.3)		116 (33.7)		88 (25.6)		25 (7.3)	
	20. How quickly did you adjust to the virtual environment experience?	3 (0.9)		2 (0.6)		11 (3.2)		41 (11.9)		133 (38.7)		86 (25)		68 (19.8)	
	21. How proficient in moving and interacting with the virtual environment did you feel at the end of the experience?	2 (0.6)		11 (3.2)		34 (9.9)		136 (39.5)		125 (36.3)		36 (10.5)		0 (0)	
	24. How well could you concentrate on the assigned tasks or required activities rather than on the mechanisms used to perform those tasks or activities?	0 (0)		1 (0.3)		4 (1.2)		59 (17.2)		131 (38.1)		118 (34.3)		31 (9)	
	25. How completely were your senses engaged in this experience?	1 (0.3)		2 (0.6)		5 (1.5)		54 (15.7)		111 (32.3)		119 (34.6)		52 (15.1)	
	27. Were there moments during the virtual environment experience when you felt completely focused on the task or environment?	2 (0.6)		0 (0)		5 (1.5)		66 (19.2)		122 (35.5)		98 (28.5)		51 (14.8)	
	28. How easily did you adjust to the control devices used to interact with the virtual environment?	0 (0)		6 (1.7)		8 (2.3)		91 (26.5)		139 (40.4)		58 (16.9)		42 (12.2)	
	29. Was the information provided through different senses in the virtual environment (eg, vision, hearing, touch) consistent?	1 (0.3)		4 (1.2)		5 (1.5)		74 (21.5)		111 (32.3)		93 (27)		56 (16.3)	
**Interface quality**															
	19. How much delay did you experience between your actions and the expected outcomes?^b^	7 (2)		32 (9.3)		64 (18.6)		102 (29.7)		57 (16.6)		58 (16.9)		24 (7)	
	22. How much did the visual display quality interfere or distract you from performing assigned tasks or required activities?^b^	17 (4.9)		79 (23)		66 (19.2)		96 (27.9)		44 (12.8)		24 (7)		18 (5.2)	
	23. How much did the control devices interfere with the performance of assigned tasks or with other activities?^b^	21 (6.1)		74 (21.5)		106 (30.8)		73 (21.2)		37 (10.8)		15 (4.4)		18 (5.2)	

^a^IVR: immersive virtual reality.

^b^Reverse items.

#### Enhanced Sympathetic Feelings Toward Older Adults With Cognitive Impairment

All participants in the focus group were impressed by the authenticity of the IVR games, which allowed them to experience the daily challenges faced by older people with cognitive impairment. One student remarked the following:

The IVR experience allowed me to see the world from the perspective of an older person with cognitive impairment who was getting lost. This experience helped me to better understand the confusion and disorientation that older people may face, which in turn helped me to be more empathetic and compassionate toward them.

Another student added the following:

This VR experience was so lifelike that it helped me to empathize with their (older people with cognitive impairment) situation and understand their needs better.

#### Improved Engagement in IVR Learning

Most participants in the focus groups said that IVR helped them stay engaged and interested in the learning process, which could sometimes be challenging in traditional classroom settings. One student said the following:

With IVR, I was able to experience the daily challenges of older adults with cognitive impairment, which made the learning process more exciting and engaging than conventional teaching methods. With this firsthand experience, I am motivated to learn and identify strategies to help them (older adults) overcome those challenges.

#### Confidence in Understanding Key Concepts in the IVR Experiential Learning Process

Students also showed confidence in their learning with IVR. They stated that learning with IVR improved their memory retention by providing a more realistic and memorable learning experience. One student commented the following:

The IVR game of delirium was a great way to simulate the condition and learn how to manage it (delirium in patients). It gave me the confidence to recognize and manage delirium in a real-life situation.

This sentiment was echoed by another student, who said the following:

The “get lost” game made me realize the importance of taking extra precautions to ensure the safety of older people with cognitive impairment. Overall, these experiences allowed me to develop a deeper understanding of the challenges associated with caring for them, which gives me more confidence in my ability to provide effective care to them.

#### Limitations of IVR Technology

Although IVR offered a unique and engaging learning experience for students, technical issues such as equipment malfunctions and slow processing times could limit the effectiveness of the IVR learning experience. One student stated the following:

I encountered some technical issues during the IVR experience, which interrupted the flow of the scenario and disrupted my immersion in the experience. It was frustrating, and I felt like I missed out on some important learning opportunities as a result.

Another student added the following:

The VR headset was heavy and its size needed to be adjusted continually to fit my head, making it difficult to fully immerse myself in the scenario. I found it challenging to stay focused and engaged during the entire experience.

### Learning Outcomes (Objective 3)

#### Overview

To understand the students’ attainment of the 3 learning outcomes after completing the IVR-assisted experiential tutorial, we conducted thematic analyses of the group written assignments. The analysis was based on 33 group assignments from the nursing students. In total, 3 themes were identified.

#### Understanding the Challenges Faced by Older People With Cognitive Impairment

The analysis of the students’ written assignments indicated that they had developed a basic understanding of the challenges faced by older people with cognitive impairment. For example, one group report stated the following:

The psychological well-being of older people would be negatively influenced due to their hallucinations. It is because restlessness and agitation would be provoked by the experiences of distorted images and sounds. The situations may happen at any time, which gives the older people much mental stress.

Another statement also said the following:

Their quality of life would be seriously affected since their cognitive functions are impaired, lowering their independence in daily living. To prevent themselves from making mistakes, they (older adults) may withdraw from society or stop doing things that they used to do. Therefore, some older adults may suffer from depression and become socially isolated due to cognitive decline.

#### Person-Centered Care

This care approach was mentioned consistently in group assignments. One report stated the following:

Person-centered care is essential to ensure that older people with cognitive impairment receive care that is tailored to their unique needs and preferences.

“Effective communication,” “family involvement,” and “supportive care with patience” were 3 critical aspects of person-centered care that were frequently discussed in the assignments:

Effective communication is a key component in person-centered care to ensure that this vulnerable group can express their needs and preferences so that the care can be tailored for them.

They also mentioned the need for family members to be included in the care planning and decision-making process. One group wrote the following:

Family members play a critical role in providing support and care to older people with cognitive impairment. This is particularly the case during delirium.

Their involvement can promote continuity of care and provide emotional support to their families with cognitive impairment, especially when they are in a distressing situation, such as delirium.

The need to be supportive was stated frequently in the written assignments. For example, one report stated the following:

As nurses, we need to provide support to individuals with cognitive impairment to promote their independence and autonomy. In order to empower them to be able to continue living their life with dignity, we should give them various forms of support.

#### Creation of an Age-Friendly Society

It was stated that this is an essential strategy to enable older people with cognitive impairment to stay in the community with dignity for as long as possible. In a written report, students recognized the need to reduce the stigma surrounding cognitive impairment and stated the following:

We need to raise awareness and educate people about the common daily challenges faced by older people with cognitive impairment to eliminate negative stereotypes and improve social inclusion for them.

Students also became aware of the importance of social inclusion in creating an age-friendly society, stating the following:

We need to create a supportive and inclusive environment that recognizes the unique needs of individuals with cognitive impairment.

They also suggested some concrete community-based services and support to enable this segment of the population to remain in their community for as long as possible. One group wrote the following:

Community-based services, such as transportation, social activities, and assistive technologies, can help them to stay connected and engaged in their communities.

Another group echoed this with the following suggestion:

Provide more community activities to enhance their interaction with the society, which can help the older adults expand their social circle to reduce the rate of deterioration of their cognitive function.

## Discussion

### Principal Findings

The results suggest that IVR-assisted experiential learning is effective in enhancing empathy toward older people among undergraduate nursing and OT students, as reflected in their higher scores on the KCES after the IVR simulation. The students reported a high level of satisfaction with the IVR learning experience, citing its suitability, ability to motivate, and innovativeness in the self-administered survey. In addition, the findings from the survey suggest that the students experienced a strong sense of presence in the IVR learning environment, enabling them to gain a deeper understanding of the challenges involved in caring for older adults with cognitive impairment. In total, 3 major themes were identified from the focus groups with 23 nursing students: *enhanced sympathetic feelings toward older adults with cognitive impairment*, *improved engagement in IVR learning*, and *confidence in understanding the key concepts through the learning process*. The thematic findings supplement and are in line with the results from the survey. The analysis of the written assignments showed that the students attained the learning outcomes of understanding the challenges faced by older people with cognitive impairment, the importance of providing person-centered care, and the need to create an age-friendly society.

These findings are consistent with those of previous studies that demonstrated the effectiveness of IVR as a mode of experiential learning to enhance the empathy of students toward older adults [49,50]. However, previous studies have mainly measured changes in students’ level of empathy using questionnaires without exploring the underlying reasons.

### Empathy Toward Older Adults and Learning Experience

Our survey findings for objectives 1 and 2 are consistent with the insights gained from the focus groups. For example, the PQ2 scores indicated that the students felt a strong sense of presence in the IVR environment, which was also reflected in their comments during the focus groups. Participants in the focus groups mentioned that the authentic IVR games allowed them to better understand and empathize with the daily challenges faced by older people with cognitive impairment, which may have contributed to the significant increase in empathy toward older adults reflected in the KCES scores. Furthermore, both the surveys and focus groups revealed that students were satisfied with the IVR-assisted experiential learning and felt confident in their ability to understand the key concepts through this approach. These consistent findings across multiple data sources provide strong evidence to suggest the effectiveness of IVR-assisted learning in enhancing students’ empathy and understanding of key concepts as well as their satisfaction with the IVR teaching approach. Compared with conventional teaching methods, IVR creates a sense of presence and provides an excellent medium for experiencing alternative points of view, allowing undergraduate health care students to virtually “step into the shoes of older adults” [23]. The hands-on experiences provided by IVR enable students to gain a deeper understanding and knowledge of the challenges that older adults with cognitive impairment may encounter [19].

The findings of this study suggest that IVR can promote positive learning experiences, including increased satisfaction, self-confidence, self-assessed competency, self-efficacy, and enjoyment among undergraduate health care students [51]. This evidence is consistent with the positive learning experiences identified in this study based on both quantitative and qualitative data. In addition, IVR facilitates a constructivist approach to education that emphasizes active participation in the learning process rather than the passive receipt of information [52]. That was why, in the focus groups, students stated that they experienced improved engagement with this innovative learning approach. It provides active and constructivist learning and increases students’ engagement in their learning, leading to an increase in the frequency of authentic learning experiences. Being engaged encourages students to become aware of learning concepts such as empathy and other soft skills needed to care for older adults.

The subgroup analysis revealed a notable enhancement in KCES scores among nursing students in contrast to a declining trend among OT students. As the aim of this study was not to draw comparisons between these 2 student groups but rather to evaluate overall empathy levels among nursing and OT students, we are unable to explain the reasons for these differences. This discrepancy could potentially be attributed to the non–discipline-specific design of the intervention, which may have been more beneficial to nursing students than to OT students.

### Learning Outcomes

Apart from enhancing empathetic experiences, an analysis of the students’ group assignments in this study revealed 3 major themes related to their learning outcomes [53]. These findings indicate that the students improved their understanding of the challenges faced by older people with cognitive impairment. Consequently, nursing students recognized the importance of person-centered care for this population, including effective communication, family involvement, and supportive care. Finally, the students highlighted the need to create an age-friendly society by reducing stigma, promoting social inclusion, and providing community-based services and support.

### Implications

By improving empathy levels through IVR experiential learning, students become more capable of comprehending needs and experiences from the perspective of the patients. The empathetic response of the students can provide insights into how newly acquired knowledge of the lived experiences of older adults with cognitive impairment can be used to enhance the quality of life of these older adults [54]. In this way, students will be better equipped to develop individualized care plans tailored to the specific needs of patients [55]. IVR experiential learning also inspires students to adopt a holistic approach when providing care to older people with cognitive impairment, recognizing the significance of social and environmental factors in their care plans [56].

### Limitations and Challenges of IVR Learning

Although IVR-assisted experiential learning has shown positive results in enhancing health care education, it is important to acknowledge the limitations and challenges associated with adopting this technology in teaching. Technical issues such as equipment malfunctions and slow processing times could result in missed learning opportunities, as noted by some students during the focus group discussions. Similar technical issues mentioned in previous studies disrupted the flow of scenarios and limited the effectiveness of the IVR learning experience [57,58]. These technical limitations must be addressed to ensure that IVR can be used effectively for teaching. Other main challenges that we experienced include the cost of implementing and maintaining the IVR technology, including hardware and software [50]. Another challenge is the need for technical support to develop and maintain IVR simulations, which requires collaboration between educators and technologists [59]. This may be prohibitive for some educational institutions to undertake.

### Study Limitations

This study had several limitations that should be considered when interpreting the results. First, without a control group for comparison, it is unclear whether the positive outcomes identified from the surveys were based solely on this teaching innovation or because of the Hawthorn effect or the effect of novelty. However, the qualitative analyses were aligned with the survey findings, providing a more comprehensive understanding of this teaching innovation. Second, the use of the self-report method may have induced expectation bias. However, anonymity was adopted when conducting the questionnaires, which may have helped minimize bias. In addition, the objective evaluation of the students’ assignments strengthened the study by providing an independent measure of their attainment of the intended learning outcomes. Third, the students’ attainment of the learning outcomes was analyzed through a group assignment; thus, we could not differentiate between individual students in terms of performance. Fourth, the study population was restricted to one undergraduate nursing and OT cohort enrolled in a single university, thereby limiting the generalizability of the findings. Fifth, we were unable to confirm the reason behind the significant difference in empathy levels between nursing and OT students as it was beyond the scope of this study. Therefore, future studies are needed to explore the specific types of IVR teaching content suitable for enhancing empathetic feelings in undergraduate students from different health care professions. Sixth, we could not confirm the transferability of the knowledge obtained through IVR-assisted experiential learning to actual clinical practice.

### Future Directions

To address the limitations of our study, we recommend conducting a randomized controlled trial with a control group in the future to evaluate the effects of IVR-assisted experiential tutorials on students’ empathy, learning experiences, and outcomes. In addition, individual assignments should be used to assess students’ attainment of the intended learning outcomes and explore factors that could affect their performance. Such a study design would allow for a more robust evaluation of the effectiveness of IVR-assisted learning and provide deeper insights into the mechanisms underlying this approach. Moreover, future studies may be needed to determine whether the designs of related interventions have to be discipline specific to enhance empathy and understanding toward older adults with cognitive impairment among students of different health care disciplines. Further observational studies in clinical areas should also be considered to explore the transferability of knowledge to clinical practice regarding IVR-assisted experiential learning.

### Conclusions

In conclusion, the findings of this study suggest that IVR-assisted experiential learning appears to have the potential to promote empathy and enhance the learning outcomes of undergraduate health care students regarding the care of older adults with cognitive impairment. Through immersive simulations, students were able to gain a deeper understanding of the challenges faced by this population and the importance of person-centered care. The findings also highlight the need to create age-friendly societies that reduce stigma, promote social inclusion, and provide community-based services and support. However, the challenges and limitations associated with the use of IVR for health care education must be addressed, such as technical issues, cost, and the need for technical support.

## Appendix

Multimedia Appendix 1 The findings on the between-group changes in Kiersma-Chen Empathy Scale scores across the time points between nursing and occupational therapy students.

## Abbreviations

IVR: immersive virtual reality
KCES: Kiersma-Chen Empathy Scale
OT: occupational therapy
PQ2: Presence Questionnaire version 2.0
VR: virtual reality

## Data Availability

The data sets are not publicly available owing to pending further analysis of the data. The virtual reality scenarios used in this study were just a part of the scenarios present in the complete virtual reality training system. As we may need to conduct further comparisons, we regret not being able to disclose the data sets.
