# Supplementary material for: The Effects of Immersive Virtual Reality–Assisted Experiential Learning on Enhancing Empathy in Undergraduate Health Care Students Toward Older Adults With Cognitive Impairment: Multiple-Methods Study
Source: JMIR Med Educ. 2024 Feb 15;10:e48566. doi: 10.2196/48566 (PMC10905348; doi:10.2196/48566)
Supplement: Multimedia Appendix 1 [file mededu_v10i1e48566_app1.pdf]

Results showing the between-group changes in KCES scores across the time points between nursing students and OT students

	Pre<br>Mean (SD)	Post<br>Mean (SD)	Time<br>(p-value)	Group<br>(p-value)	Time*Group<br>(p-value)
RS	80.16 (7.93)	77.96 (9.47)	F(1,1) = 0.143,	F(1,1) = 0.528,	F(1,1) = 13.986,
SN	77.43 (7.56)	79.23 (8.75)	p = 0.705	p = 0.468	p = 0.000***

**p < 0.05, ** P < 0.01, *** P = 0.001, KCES: Kiersma-Chen Empathy Scale*

RS: Occupational therapist students

SN: Nursing students
